# Understanding the Groundwater Hydrology of a Geographically-Isolated Prairie Fen: Implications for Conservation

**DOI:** 10.1371/journal.pone.0140430

**Published:** 2015-10-09

**Authors:** Prasanna Venkatesh Sampath, Hua-Sheng Liao, Zachary Kristopher Curtis, Patrick J. Doran, Matthew E. Herbert, Christopher A. May, Shu-Guang Li

**Affiliations:** 1 Department of Civil & Environmental Engineering, Michigan State University, East Lansing, Michigan, United States of America; 2 The Nature Conservancy, Lansing, Michigan, United States of America; University of Waterloo, CANADA

## Abstract

The sources of water and corresponding delivery mechanisms to groundwater-fed fens are not well understood due to the multi-scale geo-morphologic variability of the glacial landscape in which they occur. This lack of understanding limits the ability to effectively conserve these systems and the ecosystem services they provide, including biodiversity and water provisioning. While fens tend to occur in clusters around regional groundwater mounds, Ives Road Fen in southern Michigan is an example of a geographically-isolated fen. In this paper, we apply a multi-scale groundwater modeling approach to understand the groundwater sources for Ives Road fen. We apply Transition Probability geo-statistics on more than 3000 well logs from a state-wide water well database to characterize the complex geology using conditional simulations. We subsequently implement a 3-dimensional reverse particle tracking to delineate groundwater contribution areas to the fen. The fen receives water from multiple sources: local recharge, regional recharge from an extensive till plain, a regional groundwater mound, and a nearby pond. The regional sources deliver water through a tortuous, 3-dimensional “pipeline” consisting of a confined aquifer lying beneath an extensive clay layer. Water in this pipeline reaches the fen by upwelling through openings in the clay layer. The pipeline connects the geographically-isolated fen to the same regional mound that provides water to other fen clusters in southern Michigan. The major implication of these findings is that fen conservation efforts must be expanded from focusing on individual fens and their immediate surroundings, to studying the much larger and inter-connected hydrologic network that sustains multiple fens.

## Introduction

Fens are groundwater-fed wetlands that support the existence of a disproportionately large number of plant and animal species [1], [2]. Fens influence downstream water quantity and quality in headwater streams and wetlands [3] and buffer water temperatures as they provide cooler water in summer and warmer water in winter [4]. Fens have also been used as “whole-ecosystem gauges” of groundwater recharge, including under climate change impacts [5].

Fens are located where the right combination of climatic, hydrogeologic and topographic conditions exists [1], [6]-[9]. The unique characteristics of fens arise from the fact that they are predominantly groundwater-fed and have small surface water contributing areas [4], [10] because they are surrounded by uplands. In general, fens are known to remain saturated throughout the year but are never inundated for significant lengths of time [1], which also indicates the importance of groundwater to fen hydrology. In terms of their location on the landscape, most fens tend to be located near headwater streams [1]. Another distinguishing feature of fens is that they are known to occur in regions where the bedrock consists of limestone, dolostone and marble [1].

**Fig 1:**
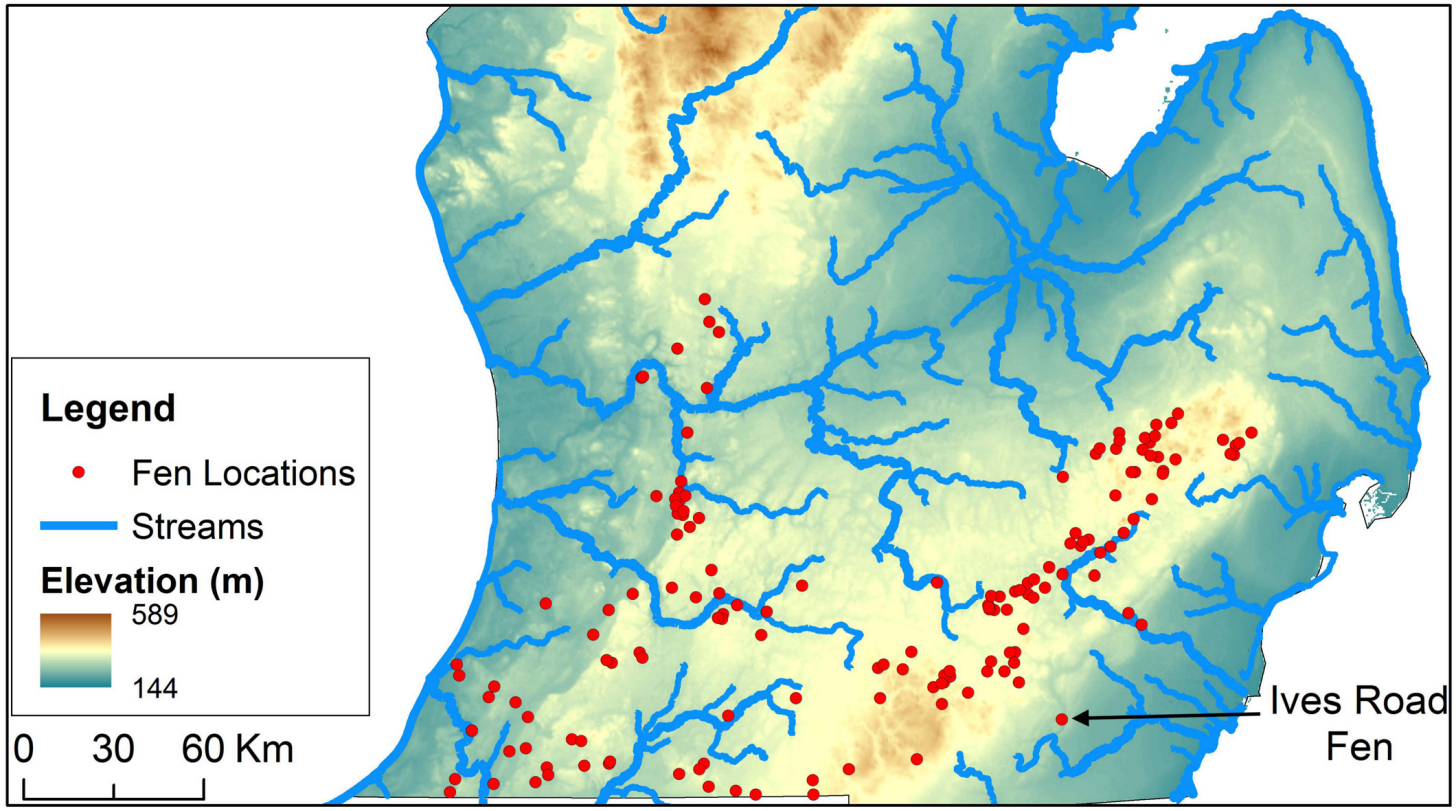
Plan view of Ives Road Fen in southern Michigan.

Where conditions are favorable, fens tend to occur in clusters [1], [2] and [11]. However, a small proportion of fens are geographically isolated from these clusters, an example of which is Ives Road Fen in Southern Michigan (Fig 1). Unlike most fens that occur in clusters, Ives Road Fen is not surrounded by uplands, but rather is placed on a topographic slope. In spite of being located on a topographic slope, the surface watershed of Ives Road Fen is rather small; therefore, surface runoff into the fen is rather limited. Moreover, the slope also ensures that the fen is never inundated by surface runoff. Also, Ives Road Fen is unique in that it is located adjacent to a 3^rd^ order stream. In addition. Ives Road fen occurs in an areas where shale is the bedrock unit. Thus, not only is Ives Road Fen geographically isolated from fen clusters, the topographic and hydro-geologic settings in which it occurs seems to be unlike those of most other fens. Therefore, understanding the sources of water and mechanisms that deliver water to this fen may provide insights into the regional and local groundwater processes that are vital to the protection and management of the fen and the many species and processes it supports.

Several studies seeking to understand the groundwater sources of fens have indicated that they benefit from multiple groundwater flow systems, both local and regional. Empirical approaches used a combination of hydraulic head, chemistry or temperature data, and hydrologic intuition to indirectly infer the existence of multi-scale flow systems that provide water to fens [11]–[19]. Several studies have stressed the need for a thorough understanding of these multi-scale flow systems that support fens [7], [20]–[22]. One of the challenges in accurately characterizing these flow systems is the lack of sufficient data to resolve the hydro-geologic variability across multiple scales.

Process-based modeling approaches used to identify the sources of water to fens have simulated flow systems either at the regional or at the local scales, ranging from relatively simple 2-dimensional models to elaborate 3-dimensional models or saturated-unsaturated flow models [23]–[27]. Vertical profile models have also been used to capture the multiple scales of flow [28], although they cannot completely characterize the 3-dimensional flow. Gilvear et al. [29]–[30] used site-specific flux and head data along with 3-dimensional models at local and regional scales and vertical profile models to understand the sources of water for fens in the United Kingdom. They inferred from upward head gradients in the regional aquifer that water from regional sources must be providing water to the fen in addition to local sources. Although they employed a regional and a refined local model, the lack of high-resolution data hampered their ability to capture the multi-scale nature of the topography, geology and hydrology (rivers and lakes).

Abbas [11] used existing groundwater and surface water data in a systematic and extensive manner to understand the groundwater hydrology of 19 fens in southern Michigan, including Ives Road Fen. The sources of water to the fens were predicted using regional groundwater flow contours. For more detailed understanding, cross-sectional models were used to predict the mechanisms that deliver water to the fens. Using this approach Abbas was able to predict local, sub-regional and regional sources that provide water to the fens in Michigan. In the case of Ives Road Fen, Abbas predicted that in addition to local recharge, the fen also obtained water from the regional Hillsdale groundwater mound, almost 20 kilometers away.

While Abbas’ [11] approach was data-driven, this research uses a more rigorous, coupled geologic modeling and process-based groundwater modeling approach to quantitatively understand the groundwater hydrology of Ives Road Fen in southern Michigan. Specifically, we 1) delineate the source water areas for the fen, 2) identify the corresponding water delivery mechanisms to the fen, 3) assess the implications of the findings on management of such fens and 4) estimate the future work required to further the understanding from this study.

## Site Description

Among the 150 fens located in Michigan, the average inter-fen distance is just over 5 kilometers. Almost two-thirds of the 150 fens have at least one adjacent fen less than 5 kilometers away. Only 10% of the fens have their nearest fen more than 10 kilometers away, of which only 3 have a neighbor 19 kilometers or farther, one of which is Ives Road Fen. This fen is located on a narrow strip of coarse-grained outwash material adjacent to the River Raisin in Lenawee County, Michigan (Fig 1). The predominant glacial material in this area is a fine-grained glacial till that occurs on both sides of the River Raisin.

### Topography and Hydrology

**Fig 2:**
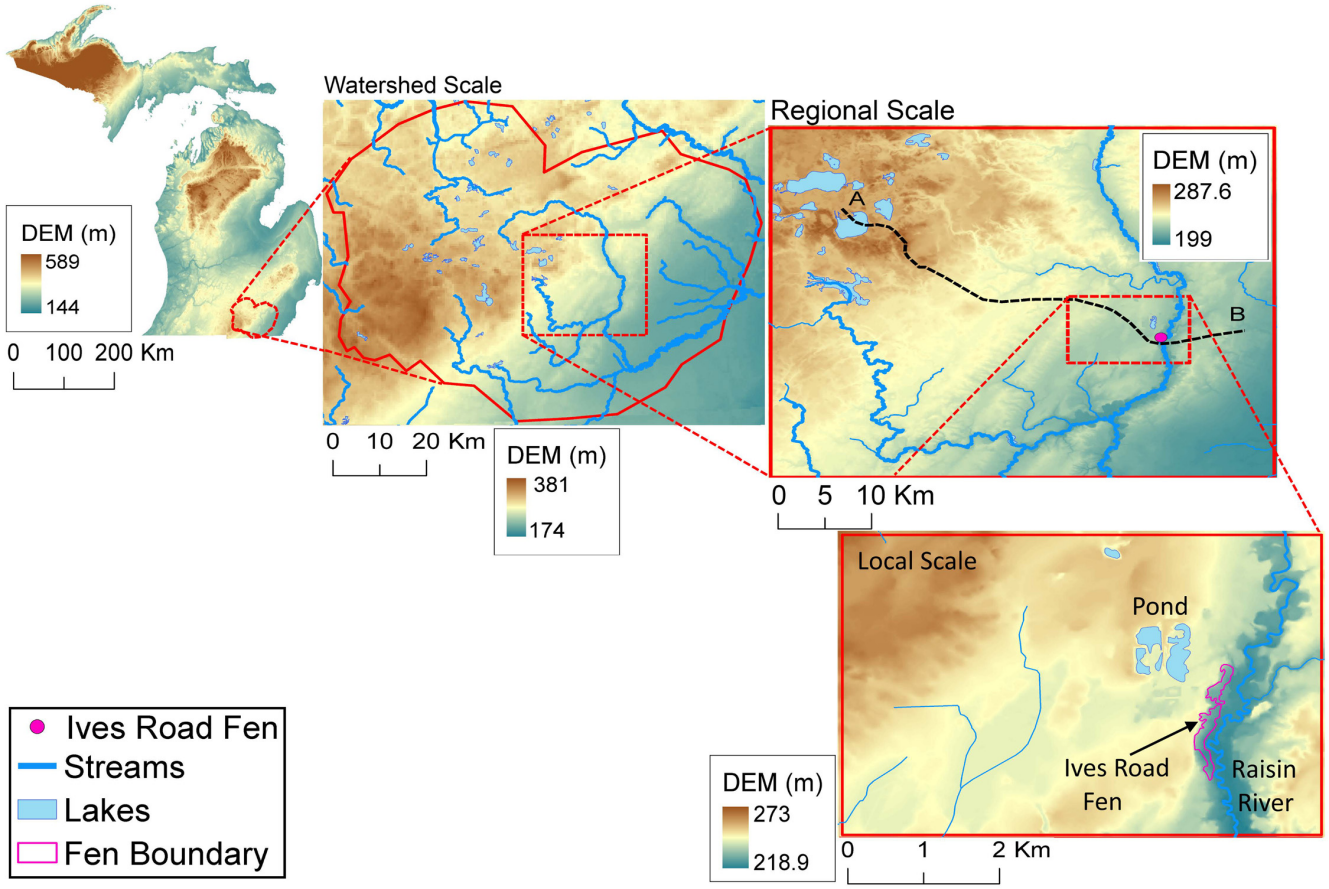
Multi-scale representation of Topography and Hydrology for Ives Road Fen.

**Fig 3:**
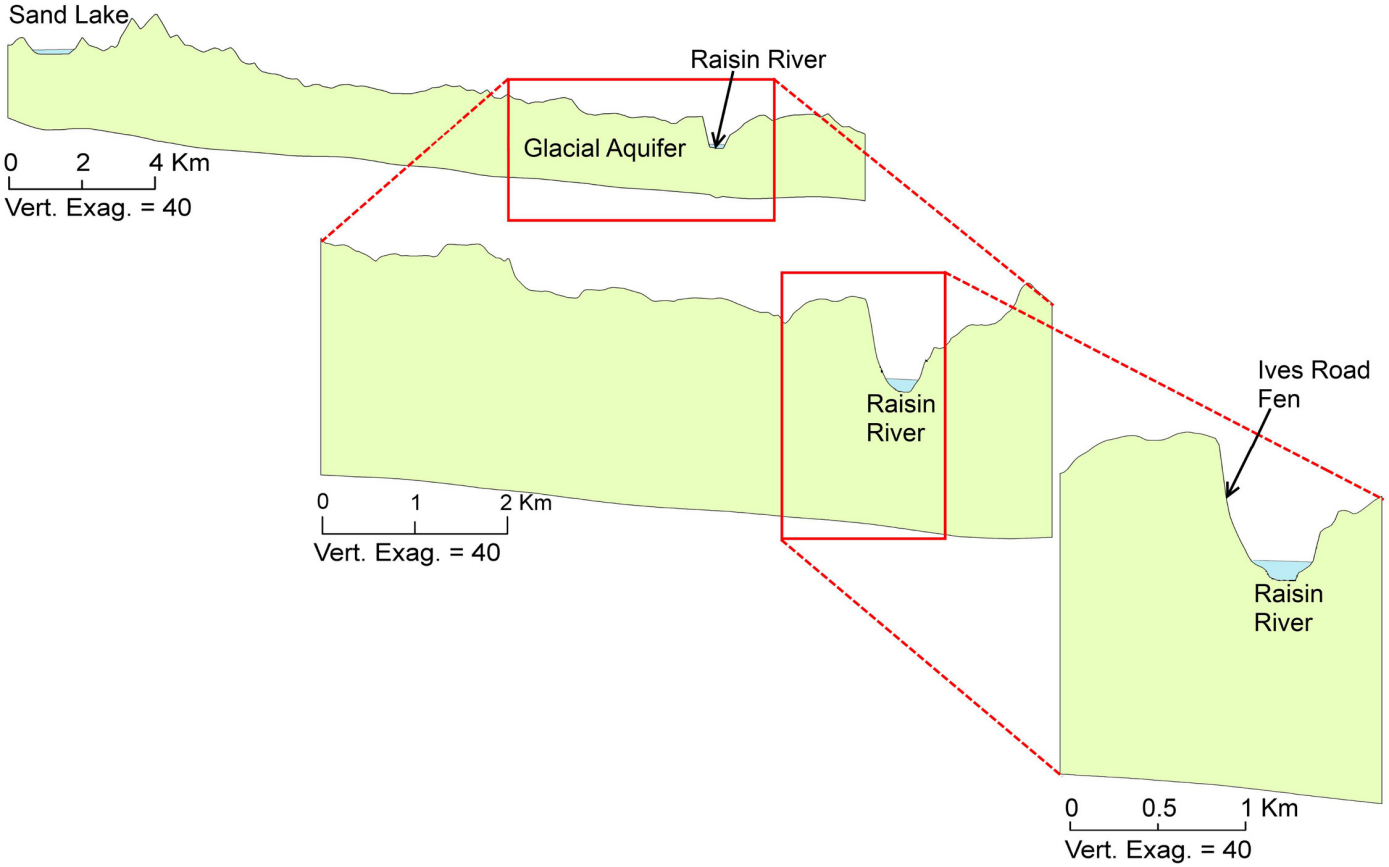
Multi-scale cross-section views of topography along cross-section AB (Fig 2).

Ives Road Fen is adjacent to two surface water bodies—the River Raisin to its east and a small pond to its northwest. The distance between the pond and the River Raisin is about 500 meters, but the difference in their surface water elevations is almost 5 meters. As a result, the land surface slopes away rather steeply from the pond towards the river, with Ives Road Fen located between the surface water bodies. Due to this slope, there is no ponding of water at the fen location, as any overland flow that may enter the fen runs off towards the River Raisin. At the regional scale, the topography slopes gently towards River Raisin from the Hillsdale mound as seen in Figs 2 and 3. Typically, in such areas where there is a general topographic slope with relatively negligible local topographic relief, regional groundwater flow systems develop [31]. Apart from River Raisin, there are no other large surface water bodies near the fen. The nearest large lakes (Lake Erin, Sand Lake, and Wamplers Lake) are almost 16 kilometers away to the west of the fen at the northeastern edge of the Hillsdale groundwater mound.

### Geology

The shallow glacial geology in a narrow band along River Raisin consists of coarse-grained deposits created by a glacial outwash channel. Under this well-sorted material, older fine-grained clay-like materials are found. Evidence for this is seen in the well logs from the statewide water well database, *Wellogic* [32]. At the regional scale, the shallow outwash aquifer is bordered on its west by a series of moraines and till plains composed of medium- and fine-textured tills. Further west is Hillsdale mound, a large glacial interlobate area composed of outwash material. The borehole data indicate that there is a fairly thick (more than 30 meters) and extensive clay layer in the till plain, beneath which a confined aquifer is found. From the borehole data we can also infer that there may be openings in the clay layer near the River Raisin caused by erosional processes of the river. These openings connect the lower confined aquifer to the shallow outwash aquifer adjacent to the River Raisin. The deeper bedrock unit in this area is the Coldwater Shale formation, which is a confining unit and can be assumed to be a no-flow boundary. The complex geologic environment in this area needs to be resolved with as much detail as possible in order to understand its impact on the fen’s hydrology.

## Methods

In order to accomplish the study objectives, a coupled geologic modeling and hierarchical, multi-scale groundwater modeling approach was used to understand the multi-scale groundwater flow system. To model the complex, 3-dimensional geology in the area of interest, a Transition Probability approach was used. In this approach, lithologic data from *Wellogic*, a state-wide well log database [32] was used to map the geologic variability. The general approach in hierarchical groundwater modeling is to model as large an area as necessary to capture the regional-scale dynamics, and then progressively refine the model in smaller areas of interest, utilizing a site-scale model(s) to resolve variability at that scale. A hierarchical patch dynamics modeling approach developed by Li and colleagues [33]–[39] that enables multi-scale modeling in a highly flexible and efficient manner was used. The geologic model was incorporated into the groundwater model to characterize the complex 3-dimensional geology in the study area. A particle tracking approach that uses the results (velocity vectors) from the groundwater models was used to delineate the sources of water and the delivery mechanisms to the fen. More details on the coupled approaches used in this study are provided next.

### Modeling complex geology

The conventional methodology in groundwater modeling is to divide the vertical extent of the model into geologic layers, such as glacial and bedrock aquifers. Lithologic data from *Wellogic* [32] indicates that using this quasi-3D approach of geologic layers cannot accurately model 3-dimensional variability in aquifer materials. In order to account for this kind of variability, a fully 3-dimensional geologic model was created using a Transition Probability approach. This geologic model was then used to inform a conceptual hydro-geologic model to predict the hydrologic connections that support the fen.

#### Transition Probability Approach

The Transition Probability approach was implemented using T-PROGS (Transition Probability Geo-Statistical Software) [40]. The first step in this approach was to classify the different geologic materials using the lithologic descriptions from the borehole data. For example, ‘Sand’ or ‘Gravel’ was classified as aquifer material (‘AQ’). Similarly, ‘Clay’ or ‘Silt’ was classified as confining material (‘CM’). Other materials that were neither ‘AQ’ nor ‘CM’, such as ‘Gravel & Silt’ or ‘Clay & Sand’ were further sub-divided into ‘MAQ’ (marginal aquifer) or ‘PCM’ (partially confining material) respectively. In principle, the borehole data may be classified into any number of such materials. For the sake of simplicity, 4 materials, ‘AQ’, ‘MAQ’, ‘PCM’ and ‘CM,’ were used to model the entire range of variability of geologic materials.

Using the above-mentioned classification scheme for the lithologic data, the geologic model was constructed using more than 3000 well logs. Using the borehole data, the transition probability matrix of auto- and cross-correlations between the different aquifer materials as a function of vertical lag spacing was created. Thus, a matrix of graphs was created, denoting the spatial variability of each aquifer material in the vertical direction, which was then fit to a geo-statistical model using a Markov chain analysis. In order to convert this vertical model into a 3-dimensional model, an anisotropy ratio, i.e., the ratio of horizontal extent of an aquifer material to its vertical extent, was assumed, which was then calibrated. More details on the transition probability approach are available in [40].

The geologic model was manually calibrated by adjusting the values of the anisotropy ratio. The vertical extent of each aquifer material was reflected in its average vertical thickness, which was inferred directly from the data. For instance, if the data showed that the average thickness of a piece of clay was 5 meters, and if the anisotropy ratio was set to 10, then the horizontal extent of the clay would be 50 meters. The geologic model was calibrated by visually comparing the simulation results to the borehole logs and to the known large-scale geologic structure. The selection of an appropriate anisotropy ratio is a rather subjective decision, which can, however, be guided by larger scale understanding of the geologic depositional processes that create spatial continuity of deposits. Carle *et al*. [41] simulated fluvial depositional systems in California using vastly different anisotropy ratios ranging from as small as 7 to greater than 400. Details of the calibrated geologic model are provided in Table 1. The calibrated values of vertical anisotropy are 15 and 8.13 for ‘AQ’ and ‘CM’ respectively, and 4 each for ‘MAQ’ and ‘PCM’. Even though the anisotropy for ‘AQ’ is almost twice as much as that of ‘CM’, the average vertical thickness of ‘AQ’ is almost 40% smaller than that of ‘CM’. Therefore, their average horizontal lengths are about the same, which is consistent with the conceptual understanding of a continuous confined aquifer below a continuous clay layer. The smaller values for ‘MAQ’ and “PCM’ also make physical sense because the proportion of these materials are relatively smaller (each less than 10%). Therefore, they are likely to have lesser spatial continuity in the lateral directions.

**Table 1:** Details of the geologic model.

Parameters	Value
NX, NY, NZ	180, 163, 117
DX, DY, DZ (m)	160.3, 159.5, 1.5
Average vertical thickness for ‘AQ’, ‘MAQ’, ‘PCM’ and ‘CM’ (m)	6.8, 5.6, 6.7, 11.5
Proportion of ‘AQ’, ‘MAQ’,’PCM’ and ‘CM’ (%)	40.8, 3.9, 8.1, 47.1
Ratio of horizontal extent to vertical thickness for ‘AQ’, ‘MAQ’, ‘PCM’ and ‘CM’	15, 4, 4, 8.13

Since each realization of the geologic model represents only one likely geologic scenario, 100 realizations of the geologic model were simulated. Ideally, each realization of the geologic model can be incorporated into the groundwater model, thus creating 100 realizations of the groundwater model. A more pragmatic approach was to incorporate the ensemble mean of the 100 realizations of the geologic model, which is an unbiased statistical representation, into the groundwater model. For example, if in a particular grid cell of the geologic model, the distribution of ‘AQ’, ‘MAQ’, ‘PCM’ and ‘CM’ over the 100 realizations was 15, 5, 5 and 75 realizations respectively, then that grid cell would be assigned ‘CM’ as the aquifer material. Importantly, this averaging procedure also made it possible to evaluate the likelihood of the openings in the clay layer discussed previously. This averaging procedure can have a significant impact on the particle tracking used to delineate the sources of water. Obviously, the particle paths predicted by the average of 100 realizations will be less dispersed than those predicted by averaging the particle paths predicted by the 100 realizations.

**Fig 4:**
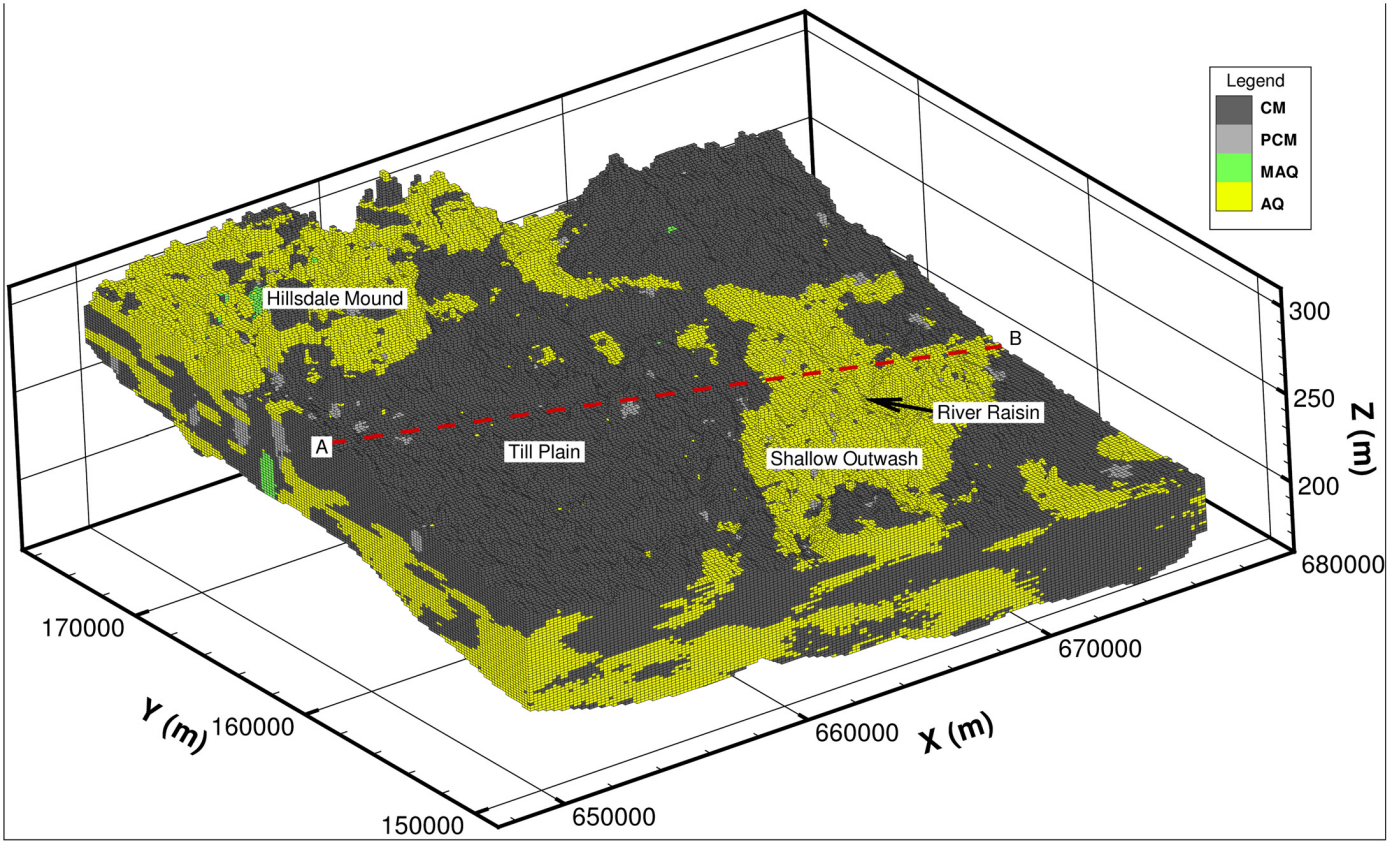
Ensemble mean of 100 realization of Transition Probability-based geologic model from lithologic data.

**Fig 5:**
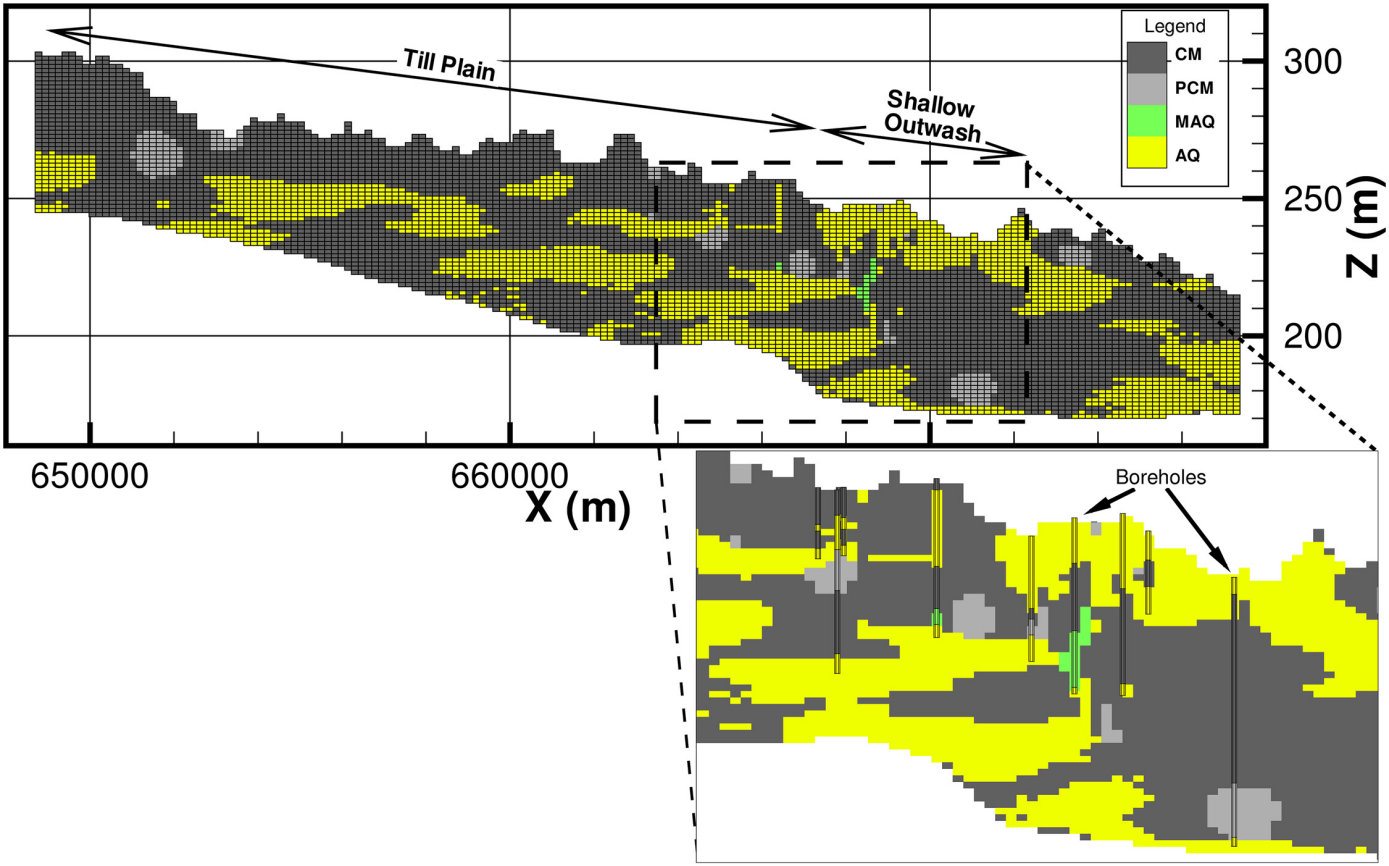
View of cross-section AB (Fig 4) from the geologic model showing the borehole data.

**Fig 6:**
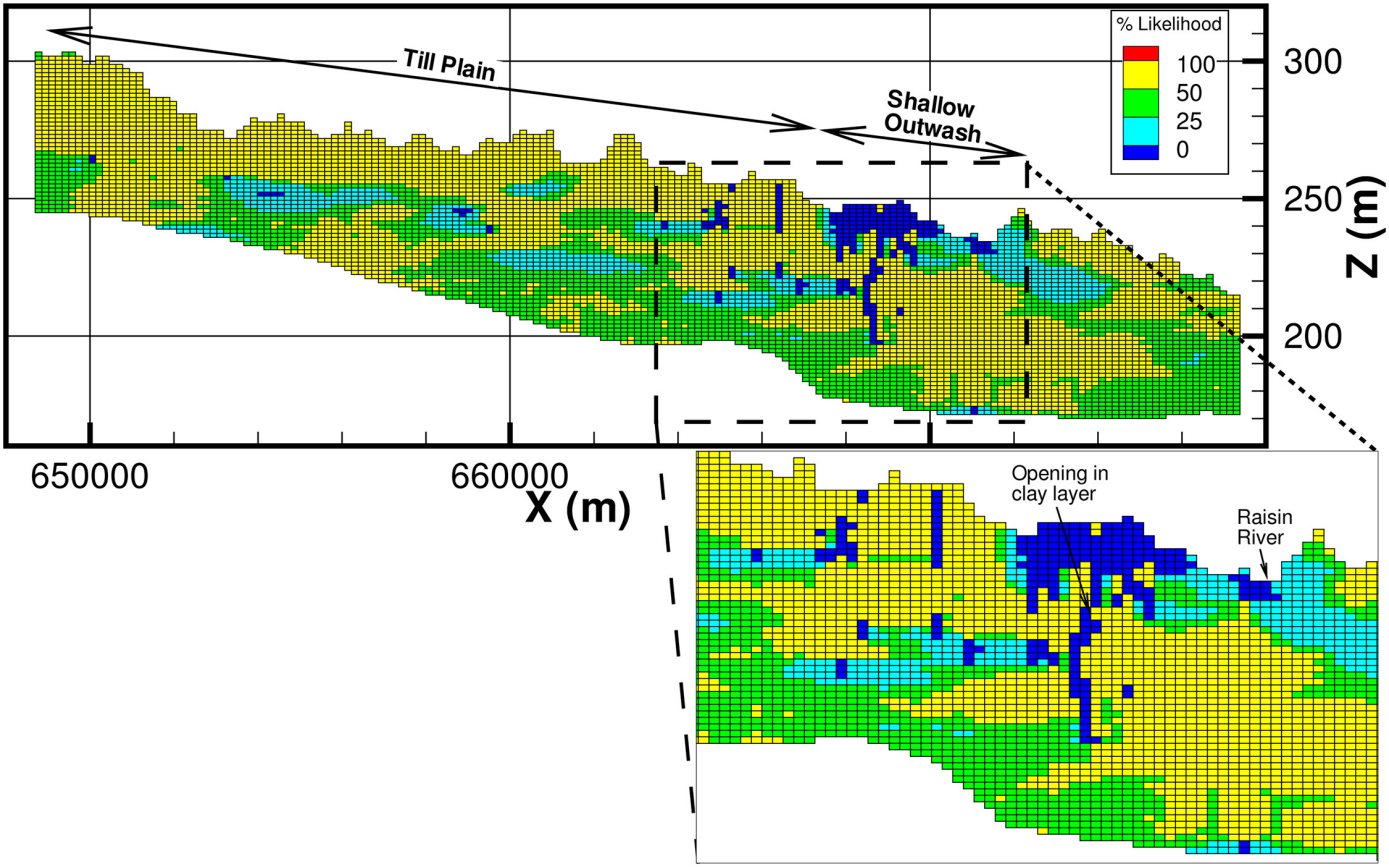
View of cross-section AB (Fig 4) from the geologic model showing the percentage likelihood of occurrence of ‘CM’ (clay) and a possible opening in the clay layer.

**Fig 7:**
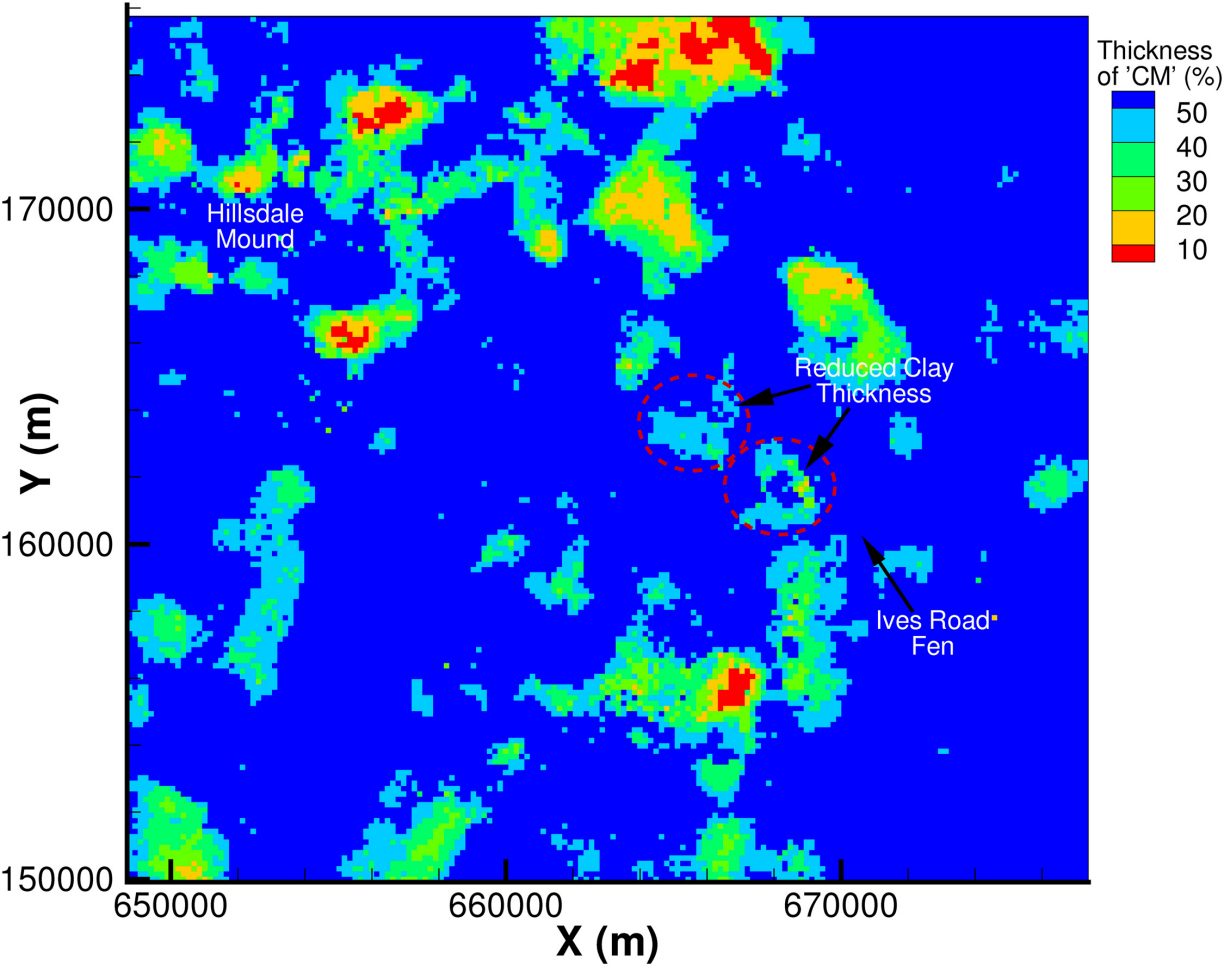
Plan view of thickness of lithologic material ‘CM” as a percentage of total aquifer thickness.

A 3-dimensional view of the ensemble mean of the 100 realizations from the geologic model is presented in Fig 4. Cross-section views of the geology and a percentage likelihood map for the occurrence of ‘CM’ along this cross-section are presented in Figs 5 and 6. In Fig 6, areas with a likelihood of occurrence of clay (‘CM’) greater than 50% are shown in yellow. From these plots it can be seen that the till plain is fairly thick and extensive as seen from the high percentage likelihood of occurrence of ‘CM’. The percentage likelihood of ‘CM’ in the shallow outwash aquifer is also extremely low (< 25%), which is also expected (low likelihood of ‘CM’ corresponds to high likelihood of ‘AQ’ and vice versa). Note that at the intersection of the till plain and the shallow outwash, there is an area of low likelihood of ‘CM’ (less than 25%), which indicates that the likelihood of an opening in the clay layer is quite high. The geologic model predicts the occurrence of ‘MAQ’ material (green cells in Fig 5) at this location, which is corroborated by the presence of a few nearby boreholes. The presence of borehole data means that this opening in the clay layer is not an artefact of the model as each realization honors the data, which also implies that the averaging procedure would have no impact on the prediction at this location. Another way to visualize this opening in the clay layer is provided in Fig 7, in which the thickness of the lithologic material ‘CM’ (clay) is shown in plan view as a percentage of the total aquifer thickness. It is evident that in a large portion of the model area the thickness of clay is greater than 50% of the total aquifer thickness (dark blue color). However, some areas near the fen (highlighted in Fig 7) show evidence of reduction in the thickness of the clay to less than 20%. This indicates that there may be “weak spots” or openings in the clay layer, which allow the deeper confined aquifer to connect to the shallow outwash aquifer. Indeed, Fogg et al. [42] suggest that high conductivity materials tend to form a “connected network”. These inter-connections may be critical in creating upwelling of water from the regional system towards the River Raisin, and potentially to Ives Road Fen.

#### Conceptual Model

**Fig 8:**
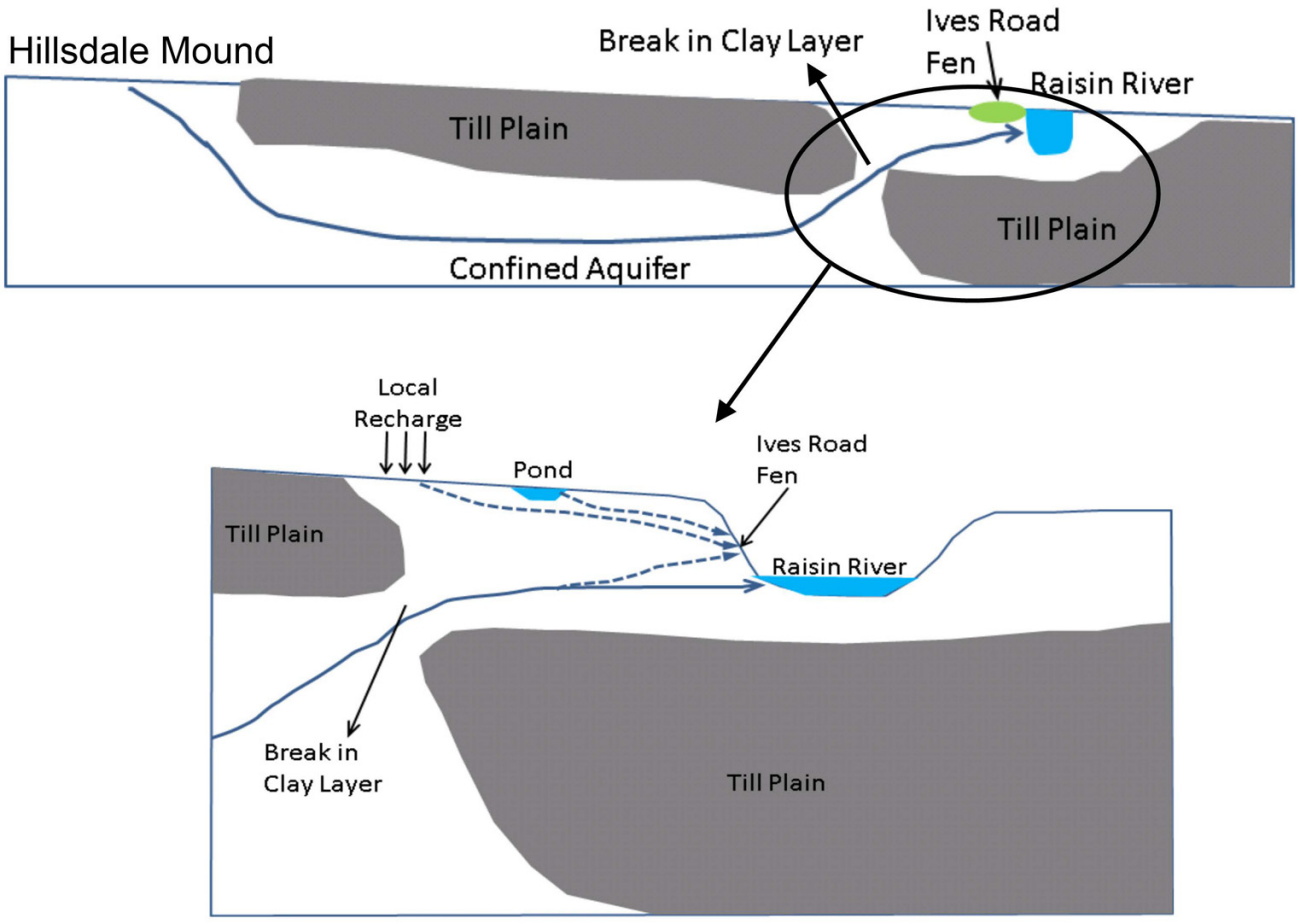
Conceptual model for Ives Road Fen.

Based on the understanding of the local and regional hydrogeologic and topographic features, it is clear that the regional discharge area is River Raisin, which must receive water from Hillsdale groundwater mound, the regional recharge area. From the 3-dimensional geologic model it is clear that water from the mound reaches River Raisin through the confined aquifer beneath the clay layer. In order to conceptualize this connection, the confined aquifer can be thought of as “pipeline” protected by the clay layer. It is important to note that this pipeline is not literally a uniform, 1-dimensional conduit but rather a tortuous, 3-dimensional preferential path created by the presence of high hydraulic conductivity materials with embedded confining materials as predicted by the geologic model. The upstream and downstream end of this pipeline are connected to the regional mound and the River Raisin respectively. This hydrologic connection to River Raisin is created by several openings in the clay layer, possibly created by erosional processes along the river. Ives Road Fen benefits from this complex geologic process. Fig 8 presents a schematic of the conceptual model for this regional connection between the Hillsdale mound and Ives Road Fen. At the local scale, the fen likely receives water from the small pond and local recharge area to the west that provide water to the shallow outwash aquifer.

### Hierarchical Groundwater Modeling

The hierarchical groundwater models were developed using Interactive Groundwater [34]–[37], an interactive modeling environment that is live-linked to GIS-enabled groundwater databases. This modeling environment allows the creation of “child” models that are dynamically coupled to their “parent” model in a recursive manner, such that a hierarchy of groundwater models is created in a flexible and easy manner. In the hierarchical groundwater modeling framework, for a generic model, *M* ^*p*,*l*^, which refers to model patch *p* in level *l*, the governing equations for steady-state groundwater flow can be given as:

$$\nabla \cdot ({\underline{\underline{K}}}^{l}\cdot \nabla H^{l})+q^{l}=0$$ (1)

With down-scaling ${H^{l}|}_{\Gamma_{1}^{l}}=f\left({H^{l-1}|}_{\Gamma_{1}^{l}}\right)$
Boundary Conditions:
Up-scaling Boundary ${H^{l}|}_{\Omega_{1}^{l}}=f\left({H^{l+1}|}_{\Omega_{1}^{l}}\right)$
Conditions:

where *H* is the hydraulic head [L], *t* is time [T], ∇ is the gradient operator, $\underline{\underline{K}}$ is the saturated hydraulic conductivity tensor [LT^-1^], *q* represents source (positive) or sink (negative) terms including pumping/injecting wells, streams, lakes, drains etc [LT^-1^]; Γ_1_ is the computational domain boundary between *M* ^*p*,*l*^ and its parent model, Ω_1_ is the computational domain of the child model, and *f* is a generic function. The superscript *l* refers to the model level for the current patch, with parent model at level *l*-1 and child model(s) at level *l*+1.

Each child or patch model in the hierarchical model derived its boundary conditions from its parent model in the form of a prescribed head boundary; this is called down-scaling. Each parent model, in turn, incorporated the finer details from its child models through up-scaling. The down/up-scaling loop was iteratively solved until a convergence criterion is met (more details in [38], [39]).

#### Groundwater Model Development

A hierarchy of steady state groundwater flow models was developed for Ives Road Fen using the data-enabled, multi-scale modeling framework, such that the multi-scale hydrologic processes were adequately resolved. Since many studies have noted that fens are characterized by saturated conditions throughout the year without being inundated for any significant length of time (i.e. a steady water table), a steady state groundwater flow model was deemed to be sufficient to simulate the fen’s conditions. Groundwater models were created at watershed, regional and local scales, which were linked to each other through an iterative two-way head coupling mechanism (see Eq 1). This mechanism consists of down-scaling in which the child models derive boundary conditions from its parent model, and up-scaling in which the parent models aggregate information from the child model to reflect local conditions. Details of the hierarchical model discretization (grid sizes and resolutions) are provided in Table 2.

**Table 2:** Details of discretization of the hierarchical groundwater model.

Model	NX	NY	NZ	DX (m)	DY (m)
Watershed Scale	207	169	4	449.0	450.2
Regional Scale	175	158	16	150.9	150.7
Local Scale	213	152	16	30.0	30.0

In areas where the topography is undulating, which the water table tends to mimic [43], dividing the aquifer thickness into multiple computational layers of equal thickness can result in “dry” cells, which can create problems in the water balance and in model convergence [44], [45]. One of the approaches to avoid this issue of dry cells is to use a coarse vertical discretization [45]. However, the complex geology in the study area necessitated the use of a fine vertical grid to resolve the geologic model. The issue of dry cells is also encountered when adjacent model cells have hydraulic conductivities varying over several orders of magnitude [46]. To overcome this issue, we used an iterative vertical discretization scheme. In this approach, the groundwater model is first discretized using one vertical layer (i.e., NZ = 1) to represent the entire aquifer thickness, and then solved to obtain the water table. In the next iteration, the water table from the previous iteration is used to sub-divide the saturated thickness of the aquifer at a finer resolution, say NZ of 2 or 4, and then solved. This process is repeated until the desired vertical resolution is achieved. As a result, the vertical grid spacing is not uniform in the model and depends on the saturated aquifer thickness at each location. This iterative process ensures that the occurrence of dry cells in the model is minimized, if not eliminated.

Topography and aquifer geometry, which form the hydro-geologic framework, are critical to the formation of local, intermediate, and regional systems of groundwater flow [31], [47]. The land surface elevation was modeled using 10 meter Digital Elevation Model (DEM) data [48]. Surface water features (lakes and streams) were incorporated from the National Hydrographic Database [49], based on the model’s spatial resolution, i.e. lakes smaller than the groundwater model’s grid size were not simulated at that scale.

The multi-scale groundwater models were conceptualized using one “conceptual” layer to represent the glacial aquifer, which was vertically discretized into multiple “computational” layers in order to resolve the heterogeneity in the glacial aquifer. The bottom of the glacial aquifer was simulated as a no-flow boundary, since the bedrock unit in this area is a confining unit (Coldwater Shale formation). The hydraulic conductivity for every groundwater model grid cell was calculated based on the proportion of the different lithologic materials (‘AQ’, ‘MAQ’, ‘PCM’ and ‘CM’) occurring within the cell using their assigned hydraulic conductivity values, which were later calibrated.

Aquifer recharge values for the groundwater model were assigned from Michigan’s Groundwater Inventory and Mapping Project [50]. The area with the extensive till plain was simulated using a calibrated lower rate of recharge, which was appropriate for an area with significant clay thickness. Surface water bodies (lakes and streams) were simulated as two-way head-dependent boundaries with leakances (which determines the ability of the lake or stream’s bed sediments to transmit water). The two-way head dependent boundary condition allows water flux to enter or leave the aquifer depending on the head gradient between the aquifer and the boundary as shown in Eq 2.

$$Q_{two-way}=L_{two-way}\{\begin{array}{ccc} \left(h_{gw}-h_{sw}\right) & if & h_{gw}>z_{bed}\\ \left(h_{sw}-z_{bed}\right) & if & h_{gw}<z_{bed} \end{array}$$ (2)

where *Q*_*two-way*_ [LT^-1^] is the flux per unit area going from the aquifer to the lake/stream or vice versa, *L*_*two-way*_ [T^-1^] is the leakance of the lake or stream, *h*_*gw*_ [L] is the head in the groundwater aquifer cell, *h*_*sw*_ [L] is the water surface elevation in the lake/stream, and *z*_*bed*_ [L] is the lake/stream’s bed elevation. Lake leakances ranged from 0.005 d^-1^ for lakes smaller than 4000 m^2^ tto 100 d^-1^ for lakes larger than 4 x 10^12^ m^2^.

Since streams are 1-dimensional features that were converted to equivalent 2-dimensional grid cells in the groundwater model, their leakance values were converted as shown in Eq 3.

$$L_{stream}=\frac{l_{stream}\cdot s_{stream}}{A}$$ (3)

where L_stream_ [T^-1^] is the effective leakance of the equivalent stream cell in the groundwater model, *l*_*stream*_ [LT^-1^] is the leakance per unit length of the stream, *s*_*stream*_ is the length of the stream in the model cell, and A [L^2^] is the area of the groundwater model cell. Stream leakances ranged from 1 md^-1^ for 1^st^ order stream to 50 md^-1^ for 6^th^ order streams.

The land surface was simulated as a one-way drain with a leakance, whose drain elevation was set to the land surface elevation. This accounted for groundwater seepage in areas where the water table elevation exceeded the land surface elevation as given in Eq 4.

$$Q_{drain}=\{\begin{array}{ccc} L_{drain}(h_{gw}-z_{drain}) & if & h_{gw}>z_{drain}\\ 0 & & otherwise \end{array}$$ (4)

where *Q*_*drain*_ [LT^-1^] is the flux per unit area leaving the aquifer through the drain, *L*_*drain*_ [T^-1^] is the leakance of the drain, *h*_*gw*_ [L] is the head in the groundwater aquifer cell, and *z*_*drain*_ [L] is the elevation of the drain. The drain leakance was set to 1 d^-1^, which was later calibrated.

#### Groundwater Model Calibration

The groundwater models were calibrated manually using static water levels from water well records in the statewide database, water level measurements at 3 locations within the fen, and base-flow estimates at USGS stream-flow gaging stations. The calibration parameters for the groundwater model were: a) multiplication factor for the aquifer recharge rate, b) recharge in the till plain, c) multiplication factors for the lake and stream leakances and the drain leakance, and d) hydraulic conductivity (*K*) values for the lithologic materials, ‘AQ’, ‘MAQ’, ‘PCM’ and ‘CM.’. The first three calibration parameters were mainly used to calibrate the groundwater model to the observed base-flow at the USGS stream-flow gauging station on the River Raisin (USGS Site 04176000). The hydraulic conductivity values were calibrated to match the hydraulic heads at the wells from the statewide database.

#### Particle Tracking

The calibrated hierarchical groundwater model was used to perform 3-dimensional, reverse particle tracking to identify the sources of water to the fen. The particle tracking algorithm was implemented within the Interactive Groundwater modeling environment [34–37], and used an Eulerian scheme to move the particles using advection only. About 120 particles were uniformly distributed in the fen area and tracked backwards through the hierarchical groundwater models in a seamless manner. Depending on whether a particle was located in the regional or local model at a particular time step, the appropriate model’s velocity field was used to move the particle to the next location. At each time step, the velocities at the particles’ location were calculated by tri-linear interpolation of the velocities from adjacent groundwater model grid nodes. This process was repeated until the particle came to a stop upon reaching a source of water (i.e., a surface water body or the water table). One of the limitations of this approach is that the particle tracking was performed in a domain in which the geologic variability was smoothed by averaging among many realizations, which can potentially under-represent the complexity of the system. It is likely that performing particle tracking with each individual realization of the geologic model would result in “dispersion” of the source area delineations compared to using a single smoothed realization. However, critical aspects of the geologic model such as the openings in the clay layer, which can have a significant impact on the particle tracking, have a much greater statistical likelihood (see Fig 6). In other words, while the lateral extent of the source area delineations may be under-estimated by the smoothed geologic realization, the connectivity between the Hillsdale mound and the fen has a significantly higher likelihood of occurrence. Also, the particle tracking approach was used only to indicate the sources of water to the fen, and not to precisely predict the flux that enters the fen or the relative contributions from the different sources delineated.

## Results and Discussion

### Hierarchical calibration

The final calibrated groundwater model parameters are presented in Table 3. During the calibration process, it was found that the hydraulic heads at the calibration locations were not very sensitive to the hydraulic conductivity values assigned to the lithologic materials ‘MAQ’ and ‘PCM’. Further examination revealed that that these two materials accounted for a small portion (about 12%) of the total aquifer materials in the geologic model (see Table 1), while the other two materials, ‘AQ’ and ‘CM’, accounted for the rest. Therefore, instead of calibrating all 4 lithologic materials, ‘MAQ’ and ‘PCM’ were treated identical to ‘AQ’ and ‘CM’ respectively, thus reducing the calibration to just 2 hydraulic conductivity parameters. A wide range of aquifer materials ranging from ‘Silty Sand’ to ‘Fine Sand’ to ‘Gravel” can potentially be characterized as ‘AQ’. The calibrated hydraulic conductivity for ‘AQ’ of 30.5 md^-1^ was within the expected range of values for these aquifer materials ranging from 10^−1^–10^4^ md^-1^ [51]. Similarly, the calibrated hydraulic conductivity for ‘CM’ of 0.3 md^-1^ was within the wide range of values expected for ‘Glacial Till’ ranging from 10^−7^–10^0^ md^-1^ [51].

**Table 3:** Calibrated model parameters.

Parameter	Value
Multiplication factor for Recharge	1.55
Recharge in the till plain (mmy^-1^)	51
Multiplication factor for lake and stream leakances	10
Multiplication factor for drain leakance	0.0001
Hydraulic conductivity ‘AQ’ (md^-1^)	30.5
Hydraulic conductivity ‘CM’ (md^-1^)	0.3

**Fig 9:**
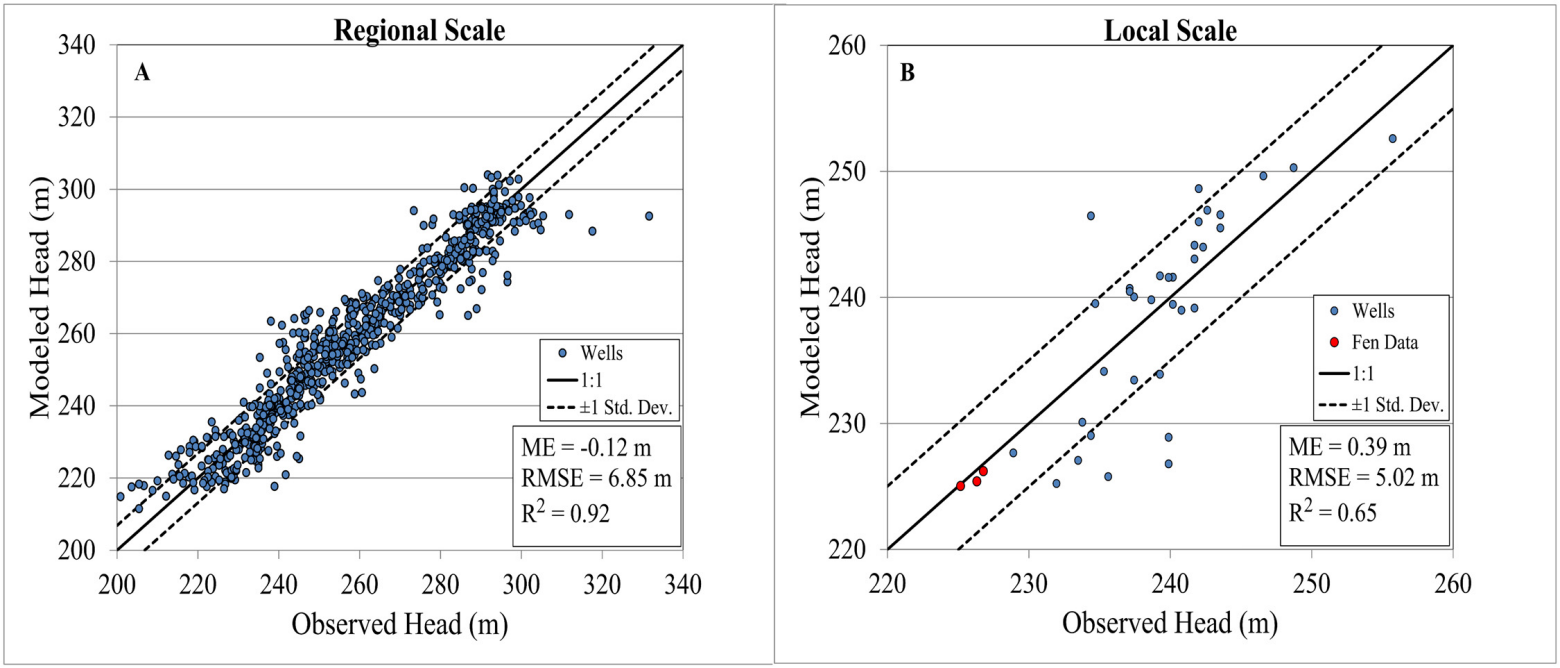
Hierarchical calibration charts for hydraulic heads in the regional (A) and local (B) models.

The base-flow into the River Raisin predicted from the groundwater model was 530,469 m^3^d^-1^, which was in agreement with the observed base-flow for the River Raisin at Adrian (USGS Site 04176000) of 538,000 m^3^d^-1^. Fig 9 shows the comparison of observed water levels with the predicted groundwater model heads for the regional and local models. At the regional scale, there were 720 data points to compare with the model, which decreased to 37 at the local scale, including 3 data points within the fen boundary. The calibration plots contain 3 indicators of the groundwater models’ performance: Mean Error (ME), Root Mean Square Error (RMSE), and the Coefficient of determination (R^2^). From the calibration plots we see that the regional scale model had an R^2^ of 0.92 while the local scale model had an R^2^ of 0.65. Even though the local scale model is able to predict the head within the fen, it is clear that the groundwater model’s performance at the regional scale is much better than at the local scale.

It is also encouraging that the relationships were highly consistent between the local and regional scales and, therefore, fitting the groundwater model through the “cloud” of data may be considered relatively satisfactory. There are, however, a number of potential factors that lead to weaker performance of the groundwater model at the local scale, including: i) poor data quality due to measurement error, temporal bias or geo-spatial inaccuracies, ii) inability to resolve local geologic features due to lack of input data, iii) inaccurate groundwater model conceptualization and iv) ineffective calibration. While the latter two factors are, in general, applicable to any model, the first two factors may have a greater impact in this case, because the data used for calibration were from the statewide database, which included data collected at various points in time across decades and during various seasons. Given that the range of head values is smaller at the local scale and the lower sample size, measurement errors can have a greater impact on the relationship with predicted values. The advantage of using these data is that the data quantity is significantly larger, especially at regional scales, than typically used in groundwater studies.

The hierarchical groundwater model was calibrated using a fairly small number of parameters, especially the complex hydraulic conductivity distribution, which was calibrated using just 2 values (hydraulic conductivity for the materials ‘AQ’ and ‘CM’). Typically, when the number of parameters is lower, the model’s ability to simulate complexity is reduced. However, in the approach used in this study, the hydraulic conductivity varied in every model cell. Relative to the simplest parameterization, i.e., a homogeneous hydraulic conductivity throughout the model, this approach was very complex; yet, it was achieved without using a large number of parameters. However, a limitation of this approach is that it assumed that the hydraulic conductivity for each aquifer material was the same throughout the model. For example, while sand located in two different areas of the model may be characterized as ‘AQ’, their hydraulic conductivity values may be similar, not necessarily identical. However, from the calibration results, capturing the geologic structure seemed to have a greater impact on the model calibration, even though intra-material variability was ignored.

### Sources of water and delivery mechanisms

**Fig 10:**
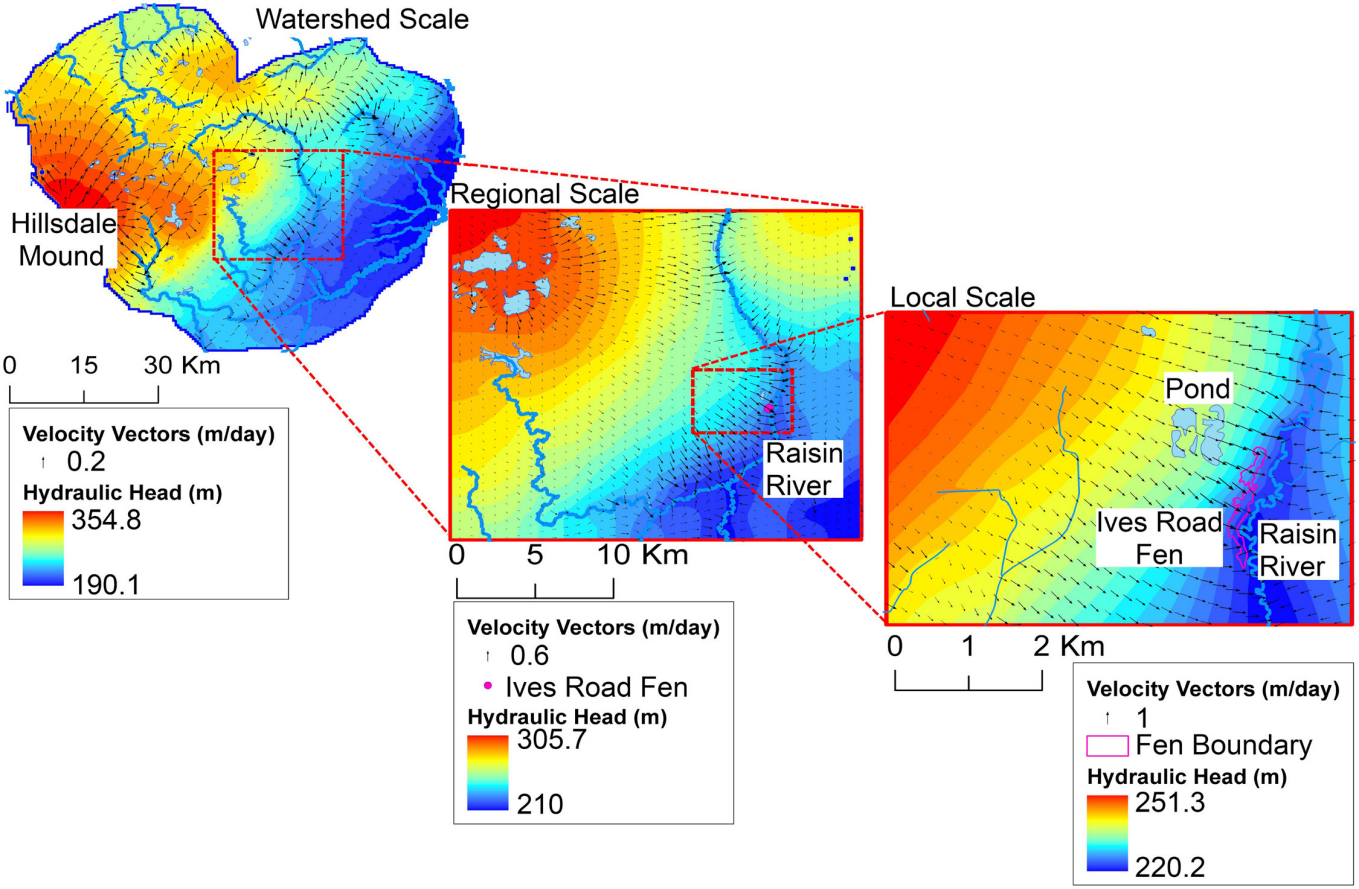
Groundwater head contours and velocity vectors from the hierarchical modeling system (1^st^ model layer).

Groundwater model simulations demonstrate groundwater flows southeast from the regional Hillsdale groundwater mound toward Ives Road Fen, with higher velocity flows near the Hillsdale mound and in the outwash near the fen, but substantially lower flow velocity throughout the Till Plain (Fig 10).

**Fig 11:**
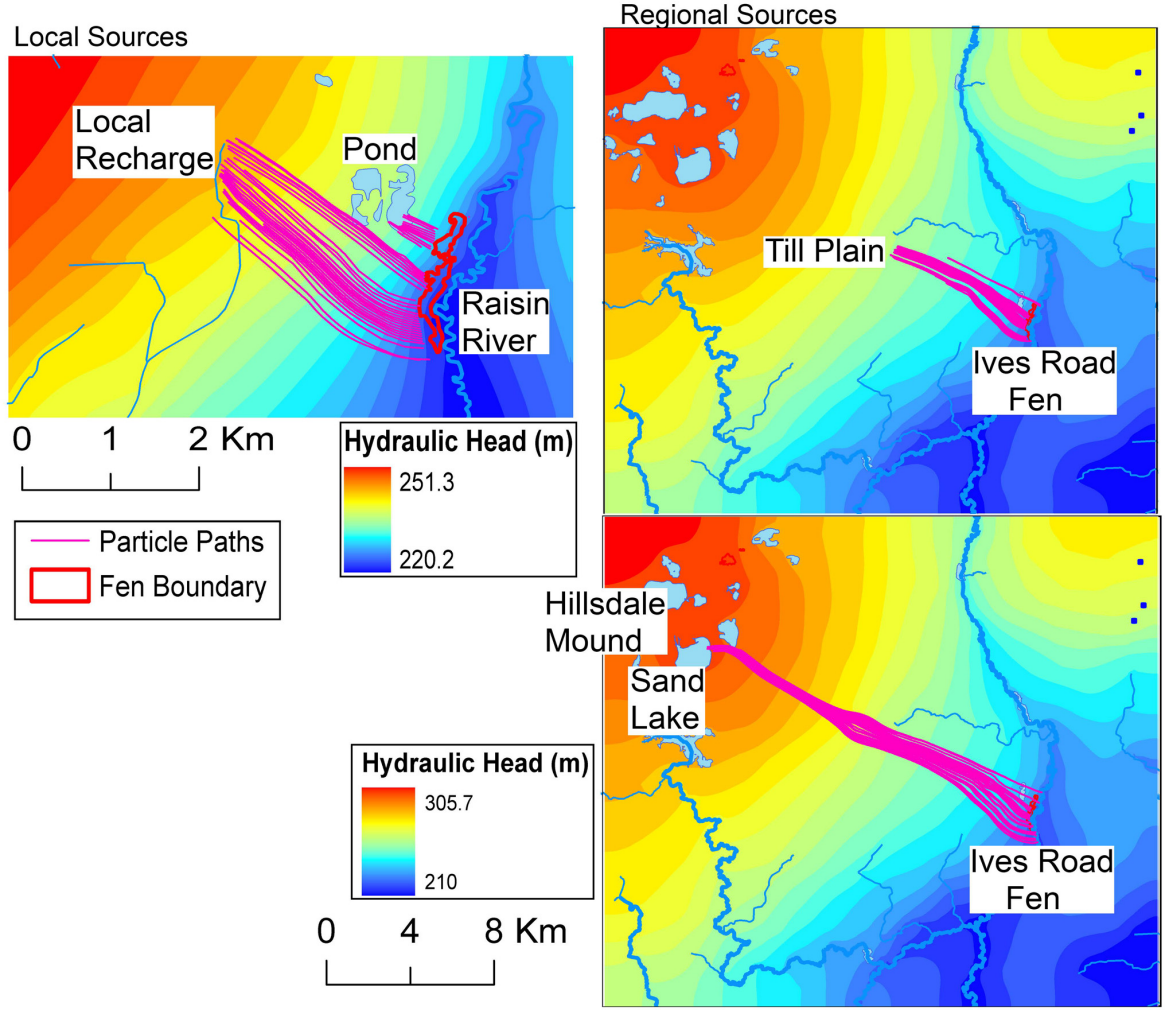
Multi-scale particle tracking results depicting sources of water to Ives Road Fen.

Using velocities from groundwater model simulations, reverse particle tracking results in Fig 11 show that Ives Road Fen is getting its water from multiple sources, including the small pond located to the west of the fen and local recharge in the shallow outwash aquifer. At the regional scale, water comes to the fen from recharge into the till plain and also from as far away as the northeastern edge of the Hillsdale groundwater mound (Sand Lake). The sources of water predicted are in agreement with the conceptual model for this site. The pond and local groundwater recharge deliver water to the fen through two distinct mechanisms, namely “direct” and “cascading” connections. Water from the local recharge area is delivered directly to the fen. On the other hand, some water from the local surface- and ground-watershed is delivered to the small pond, and then flows to the fen, and can be called a cascading connection. The regional sources deliver water to the fen through a completely different mechanism, which may be called a “pipeline” connection. Recharge from the till plain and the Hillsdale mound flows through the confined aquifer beneath the protective clay layer, and emerges through openings in this clay layer into the shallow outwash aquifer. In this area, it joins the local flow towards the River Raisin and provides water to the fen. The term “pipeline” connection is used to indicate that the clay layer forces the groundwater to travel a long distance (around 14 kilometers) in the confined aquifer before an opening in the clay layer allows it to “daylight” near the River Raisin. Therefore, contrary to the definition of Ives Road Fen as a fen “isolated” from other fen clusters, it seems to be hydrologically connected to the same regional mound that provides water to other fen clusters [11].

Of the 120 particles released at the fen, 12, 46, 38, and 24 particles tracked back to the pond, local recharge, till plain and Hillsdale mound respectively. Although these numbers cannot precisely predict the fluxes reaching the fen from the different sources, they do indicate the likely importance of the sources. Given the relatively short distance between the pond and the fen, the travel time for water from the pond to reach the fen ranged from 1 to 1.5 years, while from local recharge it ranged from 6 to 25 years. The travel time for water from the till plain to reach the fen ranged from 60 to 250 years, while from the Hillsdale mound it ranged from 250 to more than 600 years. The vastly disparate spatial and time scales of the delivery mechanisms indicate the challenges in managing such complex, inter-connected systems. Even though several studies [4], [11], [15]–[19], [21], [29] have indicated that multi-scale flow systems are very important for the survival of fens, this study is among the first to use a coupled geologic modeling and process-based groundwater modeling approach with high-resolution data to systematically demonstrate the significance of both regional and local groundwater recharge areas to a fen.

### Implications for management

The coupled geologic and groundwater modeling approach, along with the particle tracking approaches, highlight that protecting the water quantity and quality of the fen may be quite a challenging task. Local recharge seems to be the most important source of water to the fen, both in terms of the likely quantum of flux provided to the fen and the travel time for water to reach the fen; and thus, this area must be kept relatively free from hydrologic disturbances. Changing the land-use patterns in this area can cause reduction of local groundwater recharge to the aquifer, which may significantly impact the inflow to the fen. Additionally, since the shallow aquifer consists of outwash materials with high hydraulic conductivity and effective porosity, any contamination that enters the groundwater is likely to travel relatively quickly (a few years rather than decades or centuries) to the fen. Thus, land use that affects both groundwater quantity and quality in this area must be carefully managed. The small pond near the fen seems to be a relatively minor source in terms of water quantity, but it may be important during times of drought.

The other major source to the fen are the regional groundwater sources that along with local sources provide the stable, saturated soils necessary for healthy fen conditions at Ives Road Fen. The regional sources from the till plain and the Hillsdale mound are relatively robust, as they are buffered by the extensive and thick clay layer. But in order to ensure that regional sources continue to provide water to the fen, both the Hillsdale Mound and the confined aquifer that transmits water from the source to the fen need to be managed to ensure groundwater recharge of a sustainable quality and quantity. Unfortunately, areas of groundwater recharge have been shown to be underrepresented in networks of protected areas [52]. Therefore, protection of hydrologic processes for geographically isolated, but hydrologically connected, fens like Ives Road Fen will require a diversity of strategies that occur across multiple scales, such as in-field agricultural best management practices, limiting water withdrawals from the confined aquifer, and managing land-use upstream of the fen. This is important since conservation managers often evaluate protection of wetlands based, in part, on the wetland boundary (e.g., [2]), often because information on groundwater contributing areas is unavailable. If the confined aquifer is used extensively for human water supply purposes due to increased demand, this may reduce the hydraulic gradient required for water to “up-well” to the fen. Therefore, water withdrawals from this aquifer need to be regulated and monitored to maintain the hydraulic gradient needed to move water towards the fen. In terms of shallow groundwater contamination in the till plain from leaking septic tanks or underground storage tanks, the clay layer provides considerable protection by acting as a flow barrier.

## Future Work

The approach demonstrated in this study was the first step in moving towards a holistic, system-based approach to understand fen hydrology. Additionally, this study reveals the need for site-specific data that could help characterize the fen’s water balance, which is frequently absent when considering protection and management of these systems. The most important task in improving the understanding created by this study must be to quantify the fen’s mass balance and to determine the contribution of water from the different sources to the fen. Geochemical approaches have been used to trace the origins of shallow groundwater in some studies [53], and such techniques may be applied to infer the sources of water to this fen as well.

A source of uncertainty in the analysis presented in this research was the presence of openings in the clay layer. While this seemed very likely from the geologic model and from the particle tracking results, independently validating this would help remove uncertainties. One of the limitations of the methodology presented in this study was the loss of information caused by averaging the geologic model’s results to use in the groundwater model. A potential approach to overcome this would be to stochastically simulate the groundwater flow using each realization of the geologic model as input. Although such an approach is time-intensive, it can be used to map the sources of water to the fen in a probabilistic manner. Other tasks include an evaluation of the impact of climate change and other anthropogenic influences, such as land-use change, on the fen.

The current approach for fen conservation focuses on localized protection and management, rather than a system-based understanding of the underlying hydrologic system. This study illustrated that a quantitative, system-based approach is well-suited for sustainable management of fen hydrology, especially considering the potential challenges in the future as we deal with the undesirable consequences of climate change. An increase in precipitation, along with higher temperatures, milder winters and earlier springs, may have undesirable consequences on fen hydrology. For example, higher evapo-transpiration rates may cause lowering of water tables, which will cause the fens to shrink, fragment or even disappear. Anthropogenic influences, such as increased urbanization and greater demand for agricultural land, may also have a drastic impact on fens and affect their survival. The approach illustrated in this research can be used to model the effects of these perturbations, and to assess and evaluate appropriate conservation strategies for the sustainable management of fens in the future.

## Conclusions

This research presented a coupled geologic modeling and hierarchical, multi-scale groundwater modeling approach to understand the hydrologic processes that supports the persistence of fens. In particular, Ives Road Fen in southern Michigan was chosen for detailed multi-scale modeling in order to predict the sources of water and the corresponding delivery mechanisms for a geographically isolated fen. The complex geology of the study area for this research necessitated the use of a fully 3-dimensional Transition Probability approach to model the variability in aquifer materials. The results from the calibrated hierarchical simulations showed that Ives Road Fen obtains water from multiple sources, including a local recharge area, a small pond nearby, and groundwater recharge from the regional groundwater mound and a till plain. Local recharge delivers water through a direct connection and also through a cascading connection, in which water flows from local recharge to the small pond and then into the fen. Water from the regional sources is delivered to the fen through a unique pipeline connection. This pipeline consists of a confined aquifer lying below an extensive clay layer. Openings in the clay layer connect this confined aquifer to a shallow outwash aquifer, through which groundwater reaches the fen. Ives Road Fen, although seemingly isolated from other fen clusters, seems to be hydrologically connected to the same regional groundwater mound that provides water to other fen clusters. Additional data collection at the fen site and around it would further improve the understanding gained from this study.

In terms of management, it is clear that the local recharge area is vital to the fen’s survival, and needs to be protected, both in terms of groundwater quantity and quality. Given that some of the groundwater comes from regional sources, the confined aquifer also needs to be protected from large-scale water withdrawals. At the regional level, it is clear that the regional mound is a source of water to not just one fen, but to many fens, lakes, rivers, wetlands and aquifers. Thus, the regional mound is the critical node in this system, which, if protected, will have desirable outcomes for the multiple groundwater-dependent ecosystems in the region. Given the fragility of these habitats, fens should be used as barometers for the health of the overall system. Quantitative approaches, such as the one demonstrated in this research, must be used to help understand these complex ecosystems and to sustainably manage and preserve them. Findings from this study indicate the importance of understanding the connectivity of the hydrologic system across spatial and temporal scales that can be used to inform landscape-scale management.
